# Exploratory Study of rTMS Neuromodulation Effects on Electrocortical Functional Measures of Performance in an Oddball Test and Behavioral Symptoms in Autism

**DOI:** 10.3389/fnsys.2018.00020

**Published:** 2018-05-28

**Authors:** Estate M. Sokhadze, Eva V. Lamina, Emily L. Casanova, Desmond P. Kelly, Ioan Opris, Allan Tasman, Manuel F. Casanova

**Affiliations:** ^1^Department of Biomedical Sciences, School of Medicine Greenville, University of South Carolina, Greenville, SC, United States; ^2^Department of Psychiatry and Behavioral Sciences, University of Louisville, Louisville, KY, United States; ^3^Department of Pediatrics, Greenville Health System, Greenville, SC, United States; ^4^Miller School of Medicine, University of Miami, Miami, FL, United States

**Keywords:** autism spectrum disorder, TMS, ERP, reaction time, executive functions, oddball task, aberrant and repetitive behaviors

## Abstract

There is no accepted pathology to autism spectrum disorders (ASD) but research suggests the presence of an altered excitatory/inhibitory (E/I) bias in the cerebral cortex. Repetitive transcranial magnetic stimulation (rTMS) offers a non-invasive means of modulating the E/I cortical bias with little in terms of side effects. In this study, 124 high functioning ASD children (IQ > 80, <18 years of age) were recruited and assigned using randomization to either a waitlist group or one of three different number of weekly rTMS sessions (i.e., 6, 12, and 18). TMS consisted of trains of 1.0 Hz frequency pulses applied over the dorsolateral prefrontal cortex (DLPFC). The experimental task was a visual oddball with illusory Kanizsa figures. Behavioral response variables included reaction time and error rate along with such neurophysiological indices such as stimulus and response-locked event-related potentials (ERP). One hundred and twelve patients completed the assigned number of TMS sessions. Results showed significant changes from baseline to posttest period in the following measures: motor responses accuracy [lower percentage of committed errors, slower latency of commission errors and restored normative post-error reaction time slowing in both early and later-stage ERP indices, enhanced magnitude of error-related negativity (ERN), improved error monitoring and post-error correction functions]. In addition, screening surveys showed significant reductions in aberrant behavior ratings and in both repetitive and stereotypic behaviors. These differences increased with the total number of treatment sessions. Our results suggest that rTMS, particularly after 18 sessions, facilitates cognitive control, attention and target stimuli recognition by improving discrimination between task-relevant and task-irrelevant illusory figures in an oddball test. The noted improvement in executive functions of behavioral performance monitoring further suggests that TMS has the potential to target core features of ASD.

## Introduction

Autism is defined as a spectrum of behavioral disorders that share in-common impairments in social interaction and communication skills, language deficits, and a restricted repertoire of interests and stereotyped activities (American Psychiatric Association [APA], 2013). Currently, the diagnosis of autism spectrum disorder (ASD) relies on behavioral evaluations. Since there are no neuropathological findings nor laboratory tests to confirm the diagnosis, research in ASD has been guided by various conceptual models or theoretical constructs, such as executive functioning deficits (Ozonoff et al., 1991), cortical coherence weakness (Frith and Happé, 1994), information processing abnormalities (Minshew et al., 1997), theory-of-mind, empathy (Baron-Cohen, 1989, 2004), abnormal neuroplasticity (Oberman et al., 2010),“broken” mirror neuron system (Dapretto et al., 2006), altered neural connectivity (Belmonte et al., 2004; Just et al., 2004), and an alteration in the excitation-to-inhibition (E/I) bias of the cerebral cortex (Casanova et al., 2003; Rubenstein and Merzenich, 2003; Uzunova et al., 2016).

An inhibitory dysfunction model for ASD is in-keeping with reported neuropathological findings of a minicolumnopathy (Casanova, 2005, 2007; Casanova et al., 2006a,b). It also serves to explain some of the atypicality in cognitive processing, deficits in emotional reactivity, and seizures observed in autistic patients (Casanova et al., 2002a,b,c, 2014a). If this minicolumnopathy is one of the core neuropathological characteristics of ASD it would be amenable to treatment when taking into consideration the location, orientation, and nature of the neuroanatomical elements within this modular structure (Casanova et al., 2006b).

Neuromodulation and, more specifically, non-invasive brain stimulation is an intervention aimed at normalizing the putative cortical inhibitory deficit of autism. Among neuromodulation techniques, transcranial magnetic stimulation (TMS) has provided promising results as a possible therapeutic modality in many psychiatric disorders (Wassermann and Lisanby, 2001; Pascual-Leone et al., 2002; George et al., 2003; Rossi et al., 2009; Enticott et al., 2014; Rotenberg et al., 2014; Oberman and Enticott, 2015; Ni et al., 2017). Magnetic pulses generated by a current passing through a coil stimulate targeted cortical regions, usually within 2–3 cm of the skull surface (Rudiak and Marg, 1994; George et al., 1999; Hoffman and Cavus, 2002). By convention, rTMS of 1.0 Hz frequency or lower is referred to as “slow” or inhibitory, while “fast” frequencies (>1.0 Hz) as excitatory. One of the models of inhibitory rTMS effects considers long-term depression and long-term depotentiation as probable mechanisms of action (Hoffman and Cavus, 2002). It is proposed that TMS-induced alterations of cortical excitability accumulate additively with increasing number of sessions (Casanova et al., 2015). More detailed description of the biophysical aspects of TMS action can be found in Wagner et al. (2009), while basic principles of rTMS are reviewed by Klomjai et al. (2015).

Currently, TMS is used to: (1) explore stimulation-induced alterations in functional connectivity measures within and between brain regions; (2) investigate the behavioral, cognitive, and emotional relevance of these changes; and, most importantly, (3) promote changes in cortical function (Daskalakis et al., 2002). Researchers believe that these changes are achieved by inducing a temporal functional reorganization of the cerebral cortex. The rTMS-induced neuroplasticity is dependent on the state of the stimulated cortical area and on the intensity, frequency, and total number of administered magnetic pulses. Effects induced by rTMS are not limited to the stimulated cortical region, as changes are noted in other functionally interconnected areas. This makes rTMS a valuable instrument in the investigation of neuroplasticity related phenomena of the cerebral cortex (Ziemann, 2004).

The use of rTMS in autism, especially in children with ASD, has never been systematically assessed, e.g., dose, duration, type of rTMS stimulation and other variables, thus there is a need for more rigorous analysis. However, the studies of rTMS in children have not reported any notable adverse side effects. In this regard, Gilbert et al. (2004) article offered a useful reference to Institutional Review Boards (IRBs) when making a determination as to whether TMS-device based intervention poses minimal risk for treating children. The study was based on a MEDLINE review that attempted to establish any evidence of risk from TMS administration. The meta-analysis identified 28 studies involving over 850 children who underwent single or paired pulse TMS. Mild transient side effects (e.g., scalp discomfort, headaches) resolved by the day following stimulation. The authors concluded that the experimental designs of the reviewed studies conveyed no more than minimal risk to children. Similarly, the safety of TMS was confirmed in another review of more than a thousand children treated for different neuropsychiatric conditions (e.g., epilepsy, multiple sclerosis, myoclonus, ADHD, Tourette’s, and depression) (Quintana, 2005; see also Walter et al., 2001; Garvey and Gilbert, 2004; Garvey and Mall, 2008; Croarkin et al., 2011; Le et al., 2013; Hong et al., 2015; Krishnan et al., 2015).

Oberman et al. (2015) reviewed the use of TMS specifically in ASDs. Her studies covered a search in PubMed until May 2013 using TMS, autism and Asperger as keywords. The search identified 17 studies matching these keywords. From these studies, rTMS was used in 8 of them as a therapeutic tool. These 8 studies involved 104 ASD individuals. Five of these studies used the dorsolateral prefrontal cortex (DLPFC) as the site of stimulation. Side effects were minor. The authors called for more carefully designed and properly controlled studies to assess the therapeutic potential of TMS in ASD. Similar advice was provided by a consensus group on TMS application in autism research and treatment (Oberman et al., 2016).

In our own prior studies, we investigated the effects of low frequency (inhibitory) rTMS in children and adolescents with high-functioning autism and Asperger syndrome diagnosis (Sokhadze et al., 2009a,b, 2010b, 2012a,b, 2013, 2014a,b, 2016, 2017; Baruth et al., 2010a,b,c; Casanova et al., 2012, 2014b, 2015; Wang et al., 2016). Outcome measures in these studies included behavioral ratings as well as subjects’ performance (pre- and post-treatment) in a visual oddball task using reaction time measures and event-related potential (ERP) methodology. The behavioral performance findings of these studies can be summarized as group differences in response accuracy and in post-error reaction time slowing rather than in reaction time measures. We reported significant group differences in ERP measures, mostly in terms of excessive responsiveness of early ERP components (100–200 ms post-stimulus) to frequent standard and rare task-irrelevant distracter stimuli resulting in delayed late ERP potentials (200–400 ms post-stimulus) to these non-target items.

In our studies, the group of children with autism typically showed prolonged latencies of ERPs to rare novel distracters but not to targets, with this effect being observed both at the frontal and parietal topographies. In general, signs of excessive activation in the parietal cortex at the early stages of processing of task-irrelevant stimuli, along with under-activation of prefrontal cortex at the late phases of task-relevant target processing were common in children with ASD. In these studies, we could not find any abnormalities of the parietal and/or centro-parietal P3b ERP component in response to target stimuli in children with ASD as compared to typically developing children. We interpreted the results as indicating reduced discriminatory ability during performance on an oddball task in children with autism (Sokhadze et al., 2017).

The present clinical research study was designed to address some technical, feasibility, acceptability, safety and conceptual issues that were not resolved in prior published studies. Among the most important aims of the study was the comparison of three groups of total number of sessions (6 vs. 12 vs. 18), with the prediction that the higher number of TMS sessions was necessary to detect robust changes in aberrant and repetitive behaviors, targeted ERP indices, and executive functions measures manifested during performance on reaction time tests.

## Materials and Methods

### Subjects

Participants with ASD were recruited through referrals from several pediatric clinics. All patients were diagnosed according to the Diagnostic and Statistical Manual of Mental Disorders (DSM-IVTR) or DSM-5 (American Psychiatric Association [APA], 2000, 2013). Diagnosis of autism was ascertained with the Autism Diagnostic Interview – Revised (ADI-R) (Le Couteur et al., 2003). Participants were evaluated also by a developmental pediatrician. Patients had normal hearing. Patients were excluded from participation if they had a history of seizures, impairment of vision, genetic disorders, and/or brain abnormalities based on neuroimaging studies. Enrolled subjects were high-functioning children or adolescents with a full-scale Intelligence Quotient (IQ) of more than 80 according to evaluations using the Wechsler Intelligence Scale for Children, Fourth Edition [WISC-IV, (Wechsler, 2003)] or the Wechsler Abbreviated Scale of Intelligence [WASI, (Wechsler, 1999)].

The study enrolled a total of 124 children with an ASD diagnosis with 112 of them completing the assigned number of rTMS sessions. Mean age in the waitlist (WTL) group was 13.3 ± 1.78 years [*N* = 26, 4 Females (F)]; in the group of 6 sessions the mean age was 12.5 ± 1.47 years (*N* = 25, 4 F); in the group of 12 sessions the mean age was 12.8 ± 1.57 years (*N* = 30, 6 F); and in the group of 18 sessions the mean age was 13.5 ± 2.30 years (*N* = 31, 5 F). The mean age for all enrolled subjects was 13.1 ± 1.78 years (*N* = 112) without significant between-group differences. We had to exclude three participants from the 12 session TMS group and another three subjects from the 18 session TMS group (all of them boys) due to excessive artifacts and/or missing parental evaluation questionnaires, thus reducing the total number of subjects analyzed to 106 (WTL, *N* = 26; 6 TMS, *N* = 25; 12 TMS, *N* = 27, 12.9 ± 1.62 years; and 18 TMS, *N* = 28, 13.5 ± 2.31 years). Only 19 of completers were females (i.e., boys/girls ratio was ∼4.5:1). This proportion closely approximates that typically reported for gender bias in high-functioning ASD children.

Randomization at the early stages of this project followed recommendations of the local Institutional Review Board (IRB) as to assignments to either TMS or waitlist. Only after initial pilot data analysis on 9 ASD in the rTMS (6 sessions of left DLPFC) and 5 in the waitlist group and publication of results (Sokhadze et al., 2009b), we added amendment to extend session number to 12 and 18, and started randomizing after that subjects either to waitlist, 6 TMS, 12 TMS or 18 TMS.

The study was conducted in accordance with relevant national regulations and institutional policies and complied with the Helsinki Declaration. The protocol of the study including informed consent and assent forms were reviewed and received approval of the university IRB. Children and their family representatives (either parents or legal guardians) received detailed information about this research study specifics, including purpose of research, responsibilities, reimbursement rate, risk vs. benefits evaluation, etc. The participants were reimbursed only for ERP tests ($25 for each procedure), and did not receive any reimbursement for the TMS treatment. Investigators provided consent and assent forms to all families who expressed interest in participation in this treatment research study, allowed them to review these documents and answered all questions. If the child and his family member agreed to be a part of the study and confirmed their commitment, both child and parent were requested to sign and date the consent and assent forms and then received a copy co-signed by the study investigator.

**FIGURE 1:**
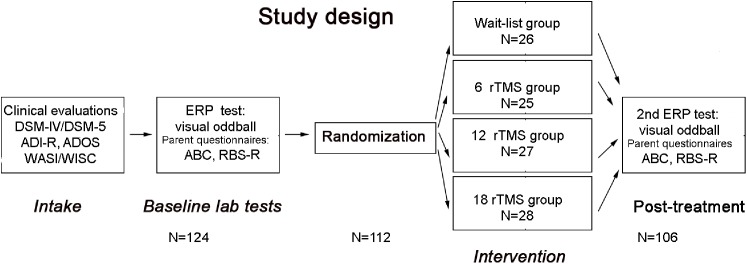
Flowchart of the study. After clinical evaluation at intake stage, subjects were tested in the lab using visual oddball task, while parents rated behavioral symptoms using ABC and RBS-R questionnaires. Then participants were randomized either to waitlist, 6 rTMS, 12 rTMS, or 18 rTMS treatment sessions and after completion of the assigned course of treatment were tested again in the lab using the same ERP test, whereas parents completed post-treatment behavioral ratings using ABC and RBS-R questionnaires.

The schematic representation of the sequence of evaluations is provided on **Figure 1**.

### Experimental Task: Visual Oddball With Illusory Kanizsa Figures

The test used in the study was a three-stimuli oddball task with rare illusory Kanizsa (Kanizsa, 1976) squares (target, 25%), rare Kanizsa triangle (novel distracter, also 25%) and frequent non-Kanizsa stimuli (standards, 50%). Visual stimuli were presented for 250 ms with inter-trial interval in 1100–1300 ms range. Before the test, all subjects had a brief practice block (20 trials only) to get familiar with the task specifics, make sure that they understood the test requirements, and that they could recognize the target stimulus correctly. There were a total of 240 trials in the study including a practice block that took around 20–25 min to complete. Participants had at least one lab visit to ensure habituation to the experimental setting and conditioning to the EEG sensor net and the lab environment.

### Behavioral Responses

Motor response measures used in the study included reaction time (RT in ms) and accuracy (percentage of correct responses) from where we calculated rates of commission and omission errors and total percentage of errors. For the calculation of post-error slowing measure, RT of the first correct response after committed error (either omission of commission error) was compared to mean RT. The difference between post-error RT and mean correct RT to target was then used to calculate normative post-error RT slowing measure (Franken et al., 2007; Sokhadze et al., 2010a, 2012a,b). Distribution of reaction time in both correct and error responses was analyzed and plotted using a sigmoid curve methodology and normalized histograms according to the technique described in Opris et al. (2016).

### Event-Related Potential Recording

The dense-array (128 channel) electroencephalogram (EEG) was recorded with an Electrical Geodesics Inc. Netstation system (EGI-Philips, Eugene, OR, United States). Experimental control (e.g., stimulus presentation, reaction time) was executed using E-prime software [Psychological Software Tools (PST), Inc., Pittsburg, PA, United States]. Visual stimuli were presented on a monitor located in front of the subject, while motor responses were recorded with a 4-bitton keypad (PST’s Serial Box). EEG was recorded with 512 Hz sampling rate, analog Notch (60 Hz, IIR) filter and analog bandpass elliptical filters set at 0.1–100 Hz range. Electrodes impedance was kept under 40 KΩ. Raw EEG recordings were segmented off-line spanning 200 ms pre-stimulus baseline and 800 ms epoch post-stimulus. EEG data was screened for artifacting purposes and all trials that had eye blinks, gross movements and other artifacts were removed using Netstation artifact rejection tools (Srinivasan et al., 1998; Luu et al., 2001). The artifact-free data for correct responses was filtered using digital Notch filter (IIR, 5th order) and also 0.3–20 Hz IIR elliptical bandpass filter. ERPs after averaging were baseline corrected (200 ms) and then re-referenced into an average reference. Commission error response-locked EEG recordings were segmented into 500 ms pre-response to 500 ms post-response. Details of our experimental procedure and EEG data acquisition, pre-processing and analysis can be found in our prior studies using the same methodology (Baruth et al., 2010a,c; Casanova et al., 2012, 2014b; Sokhadze et al., 2012b, 2014a,b).

Stimulus-locked dependent ERP variables for the frontal and fronto-central region-of-interest (ROI: F1, F2, F3, EGI channel 12, FC1, FZ, FCz, F2, F4, EGI channel 5, FC2) were N100 (80–180 ms), and P3a (300–600 ms), and for the parietal and parieto-occipital ROI (P1, P2, PO3, Pz, CPz, P3, P4, PO4) were P100 (100–180 ms) and P3b (320–600 ms) ERP components.

Response-locked ERPs dependent variables in this study were amplitude and latency of the Error-related Negativity (ERN, 40–150 ms post commission error) and Error-related Positivity (Pe, 100–300 ms post-error). The ROI for ERN and Pe components were sites between FCz and FC3- C1, between FCz and FC2-C2, and FCz).

### Transcranial Magnetic Stimulation

For repetitive TMS administration we used a Magstim Rapid device (Magstim Co., Sheffield, United Kingdom) with a 70-mm wing span figure-eight coil. To identify resting motor threshold (MT) for each hemisphere the output of the magnetic stimulator was increased by 5% steps until a 50 μV deflection of electromyogram (EMG) or a visible twitch in the First Dorsal Interosseous (FDI) muscle was detected in at least 2 or 3 trials of TMS delivered over the motor cortex controlling the contralateral FDI. EMG was recorded with a portable C-2 J&J Engineering Inc. physiological monitor with USE-3 software and Physiodata applications (J&J Engineering, Inc., Seattle, WA, United States).

**FIGURE 2:**
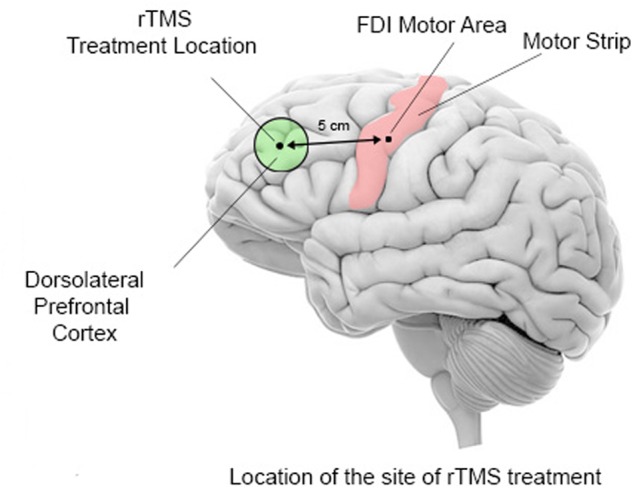
Schematic representation of TMS coil location for the stimulation of the dorsolateral prefrontal cortex. Stimulations is administered first over the left motor cortex (motor strip) to determine the optimal area for stimulation of the *first dorsal interossei* (FDI) muscle of the right hand. The output of the TMS machine is increased until the least amount of machine power that induces an EMG response or a visible twitch is identified in 4 out of 5 trials over the motor cortical area controlling the contralateral FDI. The site for rTMS treatment location is then placed 5 cm anterior to, and in a parasagittal plane to the site of maximal FDI stimulation.

In all treatment groups, the rTMS was administered on a weekly basis with the following stimulation parameters: 1.0 Hz frequency, 90% MT, 180 pulses per session with 9 trains of 20 pulses each with 20–30 s intervals between the trains. In the 6 TMS group six weekly rTMS session were administered over the left DLPFC, in the 12 TMS group 12 weekly rTMS sessions with the first six treatments were over the left DLPFC, while the next 6 were over the right DLPFC, while in the 18 TMS group the additional 6 treatments were done bilaterally over the DLPFC (evenly over the left and right DLPFC). The procedure for stimulation placed the TMS coil 5 cm anterior, and in a parasagittal plane, to the site of maximal FDI response as judged by the FDI EMG response (**Figure 2**). A swimming cap was used to ensure better positioning of the TMS coil. Positioning of the TMS coil followed recommendations that take into consideration anatomical landmarks (Mir-Moghtadaei et al., 2015; Pommier et al., 2017) and could be approximately described as the scalp region used for F3 and F4 EEG electrode placements in the 10–20 International System.

Selection of 90% of the MT was based on data from prior studies where low frequency rTMS was used for the stimulation of DLPFC in various neurological and psychiatric disorders (Pascual-Leone et al., 2000; Wassermann and Lisanby, 2001; Daskalakis et al., 2002; Gershon et al., 2003; Loo and Mitchell, 2005; Wassermann and Zimmermann, 2012). Furthermore, we decided to have stimulation power below MT as a safety precaution measure to lower the probability of seizure risk in this study population. The decision to use low frequency (below or equal 1 Hz) was based on the finding that at this frequency range rTMS exerts an inhibitory influence on the stimulated cortex (Maeda et al., 2000).

### Behavioral and Social Functioning Evaluation

Social and behavioral functioning was evaluated using caregiver (parent or guardian) reports. Participants in each group were evaluated before the TMS course (within a period of 6 weeks prestudy) and within a week following treatment. Parental reports using Aberrant Behavior Checklist [(ABC), Aman and Singh, 1994; Aman, 2004] were collected to assess irritability, lethargy/social withdrawal, stereotypy, hyperactivity, and inappropriate speech rating scores. Another parental report, specifically Repetitive Behavior Scale—Revised [(RBS-R), Bodfish et al., 1999, 2000], was used to assess stereotyped, self-injurious, compulsive, ritualistic, sameness, and restricted behaviors rating scores.

### Statistical Analysis

Repeated measure ANOVA was the primary model for statistical analyses of subject-averaged ERP, motor response and behavioral questionnaires data. Dependent behavioral variables were RT, omission and commission response rate, total accuracy, post-error RT change vs. mean correct RT, and mean RT in commission errors. Dependent stimulus-locked ERP variables were amplitude and latency of ERPs (N100, P100, P3a, and P3b) at pre-determined frontal and parietal ROIs. The within-participant factors were the following: *Stimulus* (Target Kanizsa, Non-target Kanizsa, Standard non-Kanizsa), *Hemisphere* (Left, Right), and *Time* Point (Baseline, Post-treatment). Response-locked ERPs (ERN/Pe) analysis was conducted in the same manner except using *Stimulus* and *Hemisphere* factors. The between-subject factor was *Group* (Waitlist, 6 TMS, 12 TMS, and 18 TMS). *Post hoc* analyses using Tukey and Duncan tests were conducted where appropriate. For behavioral rating scores a *Treatment* (pre- vs. post-TMS/or waiting period) factor was used. ANOVA was completed to determine changes associated with 6, 12, and 18 TMS and waitlist conditions as compared to baseline. Histograms with distribution curves were obtained for each dependent variable to determine normality of distribution and appropriateness of data for ANOVA and *post hoc t*-tests. For normality analysis we used the Shapiro–Wilk test. All dependent variables in the study had normal distribution. Greenhouse-Geisser (GG) corrected *p*-values were employed where appropriate in all ANOVAs. For the estimation of the effect size and power (Murphy and Myors, 2004) we used a Partial Eta Squared ($\eta_{p}^{2}$) and observed power computed using α= 0.05. IBM SPSS 23.0 and Sigma Stat 9.1 statistical software was used for data analysis.

## Results

### Behavioral Motor Responses (RT, Error Rate, Post-error RT)

#### Reaction Time (RT)

Effects of TMS session number [*Session* (0 in waitlist, 6 TMS, 12 TMS, 18 TMS)] on RT to targets were not significant. Comparison of RT to targets yielded no group differences in terms of session number.

#### Accuracy

**FIGURE 3:**
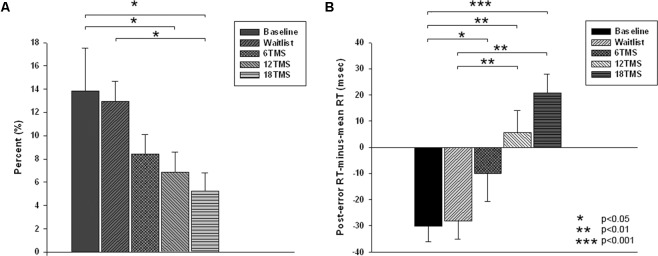
**(A)** Total error rate (in %) in oddball test at baseline, post-wait period, and post-treatment in 6 TMS, 12 TMS, and 18 TMS groups of children with ASD. Accuracy gradually improved in all TMS groups. Most significant difference was between the 18 TMS group as compared to baseline and waitlist (*p* < 0.05). Accuracy difference between three TMS groups was not significant. **(B)** Post-error reaction time (RT), calculated as first RT post-error minus mean RT, in visual oddball test at baseline and post treatment in waitlist, 6 TMS, 12 TMS, and 18 TMS groups. Most significant differences were noted between 18 TMS and baseline (*p* < 0.001), 18 TMS and waitlist (*p* < 0.001), as well as between 12 TMS and baseline (*p* = 0.004). Both 12 TMS and 18 TMS groups showed normative post-error slowing.

There were group differences in accuracy, namely in total percentage of errors [*F*_(4,207)_ = 3.77, *p* = 0.001]. Group differences in omission and commission error percentage were also statistically significant [omission errors, *F*_(4,207)_ = 2.79, *p* = 0.012; commission errors, *F*_(4,207)_ = 3.67, *p* = 0.002]. Statistical significant difference in total error rate was between 18 TMS group, waitlist and baseline (5.2 ± 8.5% in 18 TMS vs. 12.9 ± 14.3% in WTL vs. 13.5 ± 18.3% at baseline, *p*s < 0.05, see **Figure 3A** and **Table 1**). Accuracy difference between 3 TMS groups was not significant. Mean and standard deviations of reaction time, accuracy and post-error RT changes are presented on **Table 1**.

**Table 1:** Mean and standard deviation values of reaction time measures (RT, total accuracy, commission and omission rate, post-error RT change) and stimulus-licked ERN and Pe ERP components during performance on visual oddball task with illusory figures for baseline and for waitlist (*N* = 26), 6 TMS (*N* = 22), 12 TMS (*N* = 24), and 18 TMS (*N* = 23) groups.

Measures:	Baseline	Waitlist	6 TMS	12 TMS	18 TMS
Motor response and ERN/Pe	(Mean ±*SD*)	(Mean ±*SD*)	(Mean ±*SD*)	(Mean ±*SD*)	(Mean ±*SD*)
**Behavioral responses:**					
Reaction time (ms)	459 ± 102	471 ± 119	463 ± 112	473 ± 97	507 ± 82
RT in errors (ms)	348 ± 70	342 ± 82	394 ± 116	445 ± 125	452 ± 117*^†^
Total error rate (%)	13.5 ± 18.3	12.9 ± 14.3	8.2 ± 9.3	6.8 ± 10.2*	5.2 ± 8.5*^†^
Commission errors (%)	11.2 ± 17.4	8.1 ± 11.9	5.0 ± 8.1	5.6 ± 9.4	3.6 ± 7.5*
Omission errors (%)	2.3 ± 3.2	3.8 ± 5.9	3.3 ± 5.2	1.2 ± 1.8	1.6 ± 2.1
Post-error RT change (ms)	-30.6 ± 7.8	-28.1 ± 38.8	-9.8 ± 24.9*	5.7 ± 43.7**^‡^	20.7 ± 38.9***^‡^
**ERN/Pe ERP measures:**					
ERN amplitude (μV)	2.53 ± 4.98	2.27 ± 9.71	-1.49 ± 5.51	-2.05 ± 7.05	-4.47 ± 6.36*^†^
ERN latency (ms)	103 ± 44	120 ± 45	85 ± 42^†^	81 ± 44^†^	74 ± 36^†^
Pe amplitude (μV)	6.89 ± 4.61	9.41 ± 11.12	10.1 ± 9.07	9.19 ± 5.54	7.81 ± 5.78
Pe latency (ms)	199 ± 50	210 ± 42	190 ± 62	198 ± 49	190 ± 44

*Statistical significance is presented in a form of group differences with baseline (∗p < 0.05, ∗∗ p < 0.01, ∗∗∗p < 0.001) and in a form of differences between TMS groups and waitlist group (^†^p < 0.05, ^‡^p < 0.01)*.

#### Post-error RT

There were significant differences in post-error RT changes vs. mean RT, i.e., in normative post-error slowing phenomenon. Post error-RT change, calculated as the first post-error RT difference with the mean RT in correct trials, showed significant group differences [*F*_(4,207)_ = 10.03, *p* = <0.001]. Statistically significant differences were observed between 18 TMS and baseline (-61.5 ± 12.8 ms, *p* < 0.001), 18 TMS vs. WTL (-68.1 ± 13.3 ms, *p* < 0.001), as well as between 12 TMS and baseline (-48.5 ± 12.2 ms, *p* = 0.004). Both 12 TMS and 18 TMS groups showed normative post-error RT slowing (e.g., 20.6 ± 38.9 ms in 18 TMS), different from the baseline and WTL group test where post-error RT was shorter than mean RT, for instance WTL group showed -28.1 ± 38.8 ms speeding. **Figure 3B** illustrates group differences in post-error RT changes.

#### Reaction Time in Correct vs. Commission Error Responses

Main effect of response correctness on RT was significant [*F*_(1,210)_ = 58.16, *p* < 0.001]. Group differences (*Correctness* ×*Group*) were also significant [*F*_(4,207)_ = 4.06, *p* = 0.004, $\eta_{p}^{2}$ = 0.105, observed power = 0.907]. *Post hoc* analysis showed significant difference in error RT between 18 TMS group vs. baseline (*p* = 0.032) with error RT post-18 TMS being slower (452 ± 116 vs. 348 ± 70 ms).

### Posterior ERP Components (Parietal P100, P3b)

#### P100

**FIGURE 4:**
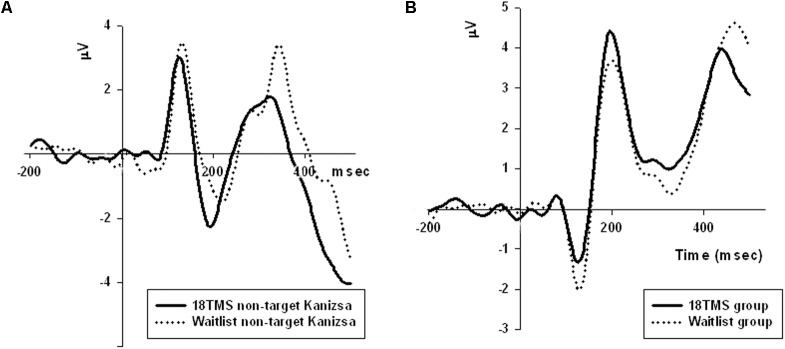
**(A)** Amplitude of P100 component at the parietal sites (ROI of five parietal channels) in response to non-target Kanizsa stimuli was significantly attenuated post-TMS, and is illustrated by comparing ERP waveforms in 18 TMS and waitlist groups. Post-TMS treatment differences (as compared to baseline and waitlist) were statistically different only for standard (*p* = 0.023) and non-target Kanizsa (*p* < 0.001) stimuli. **(B)** Latency of the frontal P3a component to non-target Kanizsa stimuli was shorter in the 18 TMS group as compared to the waitlist (*p* < 0.01), though amplitude differences were not reaching statistical significance level.

Transcranial magnetic stimulation session numbers had an effect on both amplitude and latency of parietal P100 component at all conditions (non-Kanizsa standard, non-target Kanizsa distracter, Kanizsa target). *F*-values (df = 4, 207) were in 14.8–22.36 range with all *p*s < 0.001. Amplitude of P100 post rTMS (either 6, 12, or 18 rTMS sessions) was lower and latency of P100 longer than at the baseline or in the waitlist group (*p*s < 0.01), bilaterally for standard, distracter and target stimuli. Analysis of P100 latency showed *Hemisphere* ×*Group* interaction [*F*_(4,207)_ = 5.43, *p* < 0.001, $\eta_{p}^{2}$ = 0.113, observed power = 0.972]. This effect was expressed in a prolonged latency of the P100 over the right hemisphere in all TMS groups as compared to baseline and waitlist group (**Figure 4A**). Comparison of the P100 amplitude of all three post-TMS groups as a subset showed significant differences from mean amplitude at baseline and in the waitlist group (*p*s < 0.05). Duncan test showed that 6 and 12 sessions of rTMS exerted stronger effect rather than 18 sessions and TMS group subset was not homogenous as compared to baseline and waitlist.

#### P3b

**FIGURE 5:**
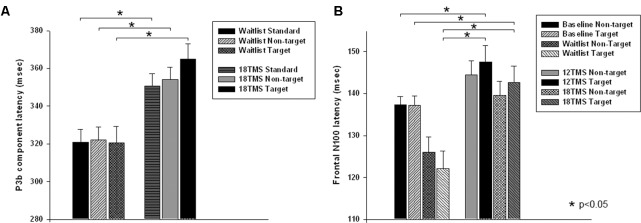
**(A)** Latency of P3b component in response to three types of stimuli (target, rare non-target, and standard) in visual oddball test in the waitlist and 18 TMS groups. Note longer latency of P3b to all stimuli, more significant in response to targets, in the 18 TMS group as compared to the waitlist group. **(B)** Latency of the frontal N100 component to non-target and target Kanizsa figures show *Stimulus* ×*Group* interaction with prolonged latencies of N100 in response to target Kanizsa as compared to non-target Kanizsa distracters in TMS groups. In particular, 12 TMS and 18 TMS groups had longer latencies to targets as compared to the waitlist group (*p* = 0.001 and *p* = 0.012, respectively).

**FIGURE 6:**
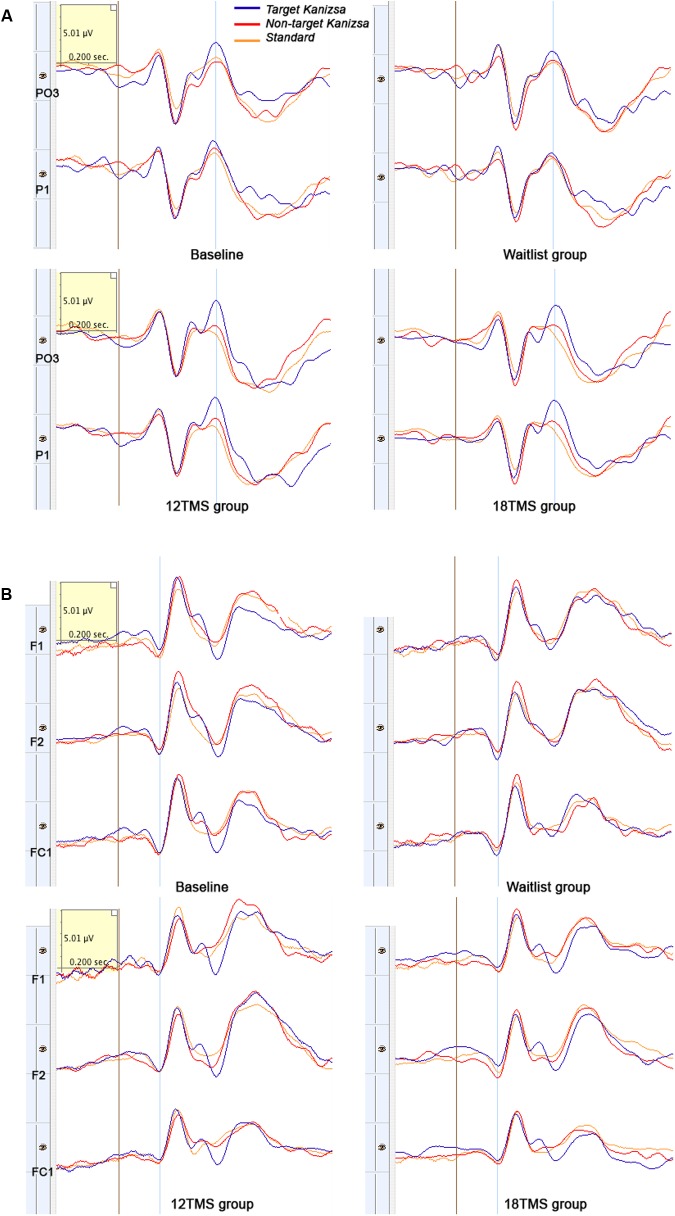
**(A)** Left parieto-occipital (PO3) and parietal (P1) ERPs to target Kanizsa, non-target Kanizsa and non-Kanizsa standard stimuli at baseline, and in waitlist, 12 TMS and 18 TMS groups. Both 12 TMS and 18 TMS groups show lower amplitude of the P3b components (marked by blue line) to non-target stimuli. In the waitlist group ERPs to all three types of stimuli are comparable by their P3b amplitude, especially at the P1 site. **(B)** Frontal (F1, F2) and fronto-central (FC1) ERPs to three types of stimuli in oddball task at baseline and in waitlist, 12 TMS, and 18 TMS groups. Amplitude to all types of stimuli post-TMS treatment was decreased, more in the 18 TMS group. Note delayed latency and higher amplitude of the P3a component in response to non-target Kanizsa distracter in the waitlist group.

Stimulus type (standard, distracter, and target) had main effect on P3b amplitude [*F*_(2,209)_ = 35.72, *p* < 0.001] and P3b latency [*F*_(2,209)_ = 15.61, *p* < 0.001], with amplitude being higher and latencies longer in response to targets as compared to standards and distracters. This difference was statistically significant when post-18 TMS and waiting group P3b amplitude and latencies were compared (respectively, *p* = 0.016 and *p* = 0.004). Main effect of group factor was significant both for amplitude and latency (*p*s < 0.001). The latency of P3b yielded *Stimulus* × *Group* interaction [GG-corrected *F*_(8,194)_ = 3.19, *p* = 0.003, $\eta_{p}^{2}$ = 0.066, observed power = 0.851], expressed as bilaterally longer latency to targets rather than to standard and distracter stimuli in the 18 TMS group as compared to the waitlist group (*p* = 0.004). Similar effect was observed as well in the 6 TMS group (*p* = 0.006). Post-treatment P3b amplitude group differences were significant for standard (*p* = 0.023) and distracter (*p* < 0.001) but not target (*p* = 0.11) stimuli, though group differences were significant for P3b latency for all type of stimuli (see **Figures 5A**, **6A** and **Table 2**). Post-12 TMS and 18 TMS amplitude of P3b was lower and latency longer to both standards and distracters as compared to baseline and waitlist (all *p*s < 0.05). Latency of P3b post-18 TMS was prolonged as compared to waitlist (difference was 33.3 ± 9.4 ms, *F* = 3.01, *p* = 0.004) bilaterally. Hemispheric group differences were not significant for both latency and amplitude of P3b component.

**Table 2:** Mean and standard deviation values of amplitude and latency of the parietal P3 and P100 ERP components and latencies of the frontal P3a and N100 ERP components for baseline and for waitlist (*N* = 26), 6 TMS (*N* = 25), 12 TMS (*N* = 27) and 18 TMS (*N* = 28) groups.

ERP measures:	Baseline	Waitlist	6 TMS	12 TMS	18 TMS
Amplitude (μV) and latency (ms)	(Mean ±*SD*)	(Mean ±*SD*)	(Mean ±*SD*)	(Mean ±*SD*)	(Mean ±*SD*)
**Parietal (bilateral):**
P3b amplitude standard	3.97 ± 3.08	5.19 ± 3.29	3.64 ± 4.59	3.02 ± 2.20	2.44 ± 3.03^†^
P3b amplitude distracter	4.29 ± 3.42	5.80 ± 3.88	2.72 ± 4.35	2.94 ± 2.07*^†^	2.44 ± 3.02*^†^
P3b amplitude target	6.00 ± 4.85	6.07 ± 4.36	5.54 ± 5.92	4.12 ± 2.71	4.26 ± 4.13
P3b latency standard	340 ± 30	323 ± 20	327 ± 37	336 ± 41^†^	351 ± 47^†^
P3b latency distracter	342 ± 30	324 ± 22	351 ± 37^†^	338 ± 32^†^	352 ± 50^†^
P3b latency target	343 ± 42	321 ± 27	359 ± 42*^†^	348 ± 89*^†^	362 ± 56*^†^
P100 latency standard	136 ± 27	133 ± 17	144 ± 31	145 ± 39^†^	149 ± 33*^†^
P100 latency distracter	134 ± 30	130 ± 20	147 ± 36	149 ± 39*^†^	151 ± 32*^†^
P100 latency target	135 ± 29	128 ± 18	146 ± 35	156 ± 35*^†^	149 ± 34*^†^
**Frontal and frontocentral:**					
P3a amp. standard right	4.53 ± 4.73	5.35 ± 3.96	2.93 ± 3.50	2.78 ± 2.27^†^	4.12 ± 2.72
P3a amp. distracter right	5.67 ± 4.79	6.33 ± 5.32	1.62 ± 3.74*^‡^	2.41 ± 2.40*^†^	3.32 ± 3.52
P3a amp. target right	6.02 ± 4.61	5.67 ± 4.42	4.17 ± 5.41	3.76 ± 3.14	4.10 ± 4.04
N100 latency standard	135 ± 21	130 ± 12	138 ± 16	142 ± 12	142 ± 25
N100 latency distracter	135 ± 20	123 ± 15	138 ± 21	147 ± 22^†^	141 ± 24^†^
N100 latency target	136 ± 21	120 ± 19	138 ± 19	145 ± 18^†^	143 ± 28^†^

*Statistical significance is presented in a form of group differences with baseline (∗p < 0.05, ∗∗p < 0.01, ∗∗∗p < 0.001) and differences between TMS groups and waitlist group (^†^p < 0.05, ^‡^p < 0.01).*

### Anterior ERP Components (Frontal N100, P3a)

#### N100

Stimulus type had no main effect on frontal N100 amplitude. Analysis of *Stimulus* and *Hemisphere* factors effects on N100 latency yielded both *Stimulus* ×*Group* [*F*_(8,414)_ = 2.03, *p* = 0.042, $\eta_{p}^{2}$ = 0.040, observed power = 0.825] and *Hemisphere* × *Group* [*F*_(4,207)_ = 40.28, *p* < 0.001, $\eta_{p}^{2}$ = 0.287, observed power = 0.991] interactions. These interactions can be described as prolonged latencies of N100 in response to target Kanizsa as compared to non-target Kanizsa distracters in TMS groups with effect being more pronounced at the right hemisphere. In particular, 12 TMS and 18 TMS groups had longer latencies as compared to the waitlist group (*p* = 0.001 and *p* = 0.012, respectively). For instance, N100 latency to targets over the right frontal and fronto-central ROI was significantly shorter in waitlist as compared to 12 TMS and 18 TMS groups (123.4 ± 14.6 ms in WTL, 147.2 ± 41.5 ms in 12 TMS, and 142.4 ± 23.5 ms in 18 TMS, all *p*s < 0.05). Comparison of latency of N100 ERP component in TMS and waitlist groups waitlist showed prolongation to target and shortening in response to non-target Kanizsa figures in the TMS groups, whereas latency of N100 in all conditions remained the same in the WTL group (**Figure 5B** and **Table 2**). We could not find any statistically significant group differences in N100 amplitude.

#### P300 (P3a)

*Stimulus type* (standard, distracter, and target) bilaterally had main effect on P3a amplitude [*F*_(2,209)_ = 7.19, *p* = 0.001] and on P3a latency [*F*_(2,209)_ = 6.19, *p* = 0.002].

Group differences in amplitude were significant only for non-target stimuli type at the right hemisphere, specifically for standards (*F* = 4.18, *p* = 0.003) and non-target Kanizsa distracters (*F* = 7.62, *p* < 0.001, **Figure 4B**). However, latency of P3a showed group differences in response to all three conditions at both hemispheres (all *p* < 0.01). Latency of P3a showed significant *Hemisphere* ×*Group* interaction [*F*_(4,207)_ = 23.77, *p* < 0.001, $\eta_{p}^{2}$ = 0.334, observed power = 0.992]. The interaction was characterized by lower latency of P3a at the right hemisphere in the TMS groups as compared to waitlist group. Both 12 TMS and 18 TMS groups had shorter latencies to non-target Kanizsa distracters as compared to waitlist group (WTL, 396 ± 41 ms; 12 TMS, 348 ± 35 ms; 18 TMS, 379 ± 42 ms, *p*s < 0.01). Similar effect was observed for standards in these two TMS groups (*p*s < 0.01) but not for target Kanizsa stimuli (**Figure 6B**).

### Response-Locked ERPs (Frontal and Fronto-Central ERN/Pe)

Since eight subjects showed insufficient number of commission errors, they were excluded in the statistical analysis. Effects of group factor on ERN amplitude [*F*_(4,199)_ = 3.75, *p* = 0.007] and ERN latency (*F* = 5.54, *p* = 0.001) were found to be statistically significant. *Post hoc* analysis showed that the amplitude of ERN in post-18 TMS group was more negative and significantly different both from both baseline (by -7.00 ± 2.30 μV, *p* = 0.023) and waitlist group (-8.74 ± 2.97 μV, *p* = 0.032). Latency of ERN post-18 TMS was shorter than in the waitlist group (45.9 ± 14.9 ms, *p* = 0.022). There were no group differences in amplitude and latency of the Pe component.

### Clinical Behavior Evaluations Post- TMS

#### Repetitive Behavior Scale Outcomes

**FIGURE 7:**
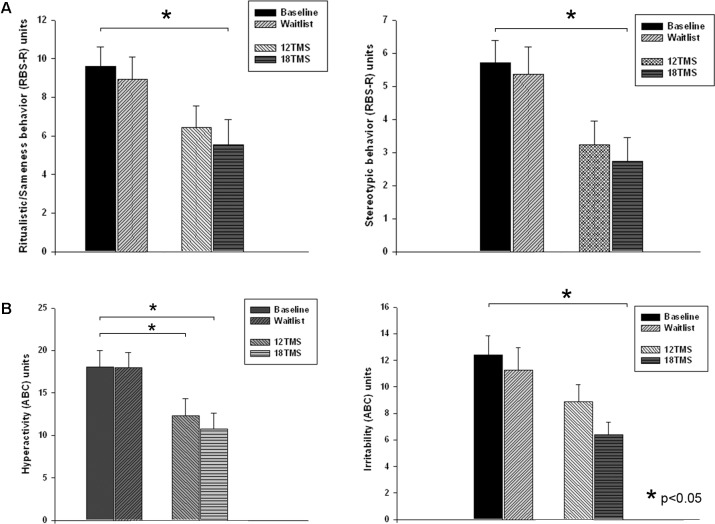
**(A)** *Ritualistic/Sameness* behavior (left) and *Stereotype* behavior (right) rating scores of RBS-R questionnaire at baseline, post-waiting period, and post 12 and 18 sessions of rTMS. Most dramatic decrease of scores was observed in the 18TSM group. **(B)** *Hyperactivity* (left) and *Irritability* (right) rating scores of ABC questionnaire at baseline, post-waiting period, and post 12 and 18 sessions of rTMS. The *Irritability* scores in the 18 TMS group decreased practically in half (–6.01, *p* = 0.029) as compared to the baseline.

Repetitive behavior subscales (RBS-R, Bodfish et al., 1999) showed group difference for *Stereotype Behavior* [*F*_(4,205)_ = 2.68, *p* = 0.035, see **Figure 7A**] and *Total Repetitive Behaviors T-score* [*F*_(4,205)_ = 3.26, *p* = 0.014]. *Post hoc* analysis showed significant lower *T-score* of RBS-R in 18 TMS group as compared to baseline (14.6 ± 12.8 vs. 26.5 ± 15.2, *p* = 0.014). *Ritualistic Behavior* rating decreased from baseline in the 18 TMS group (from 9.61 ± 6.00 down to 5.55 ± 6.18 post-treatment, *p* = 0.017, **Figure 7A**, left). *Stereotype Behavior* rating in this TMS group also decreased as compared to baseline (from 5.71 ± 4.20 down to 2.73 ± 3.29, *p* = 0.037, **Figure 7A**, right).

#### Aberrant Behavior Checklist Outcomes

Three of the ABC (Aman and Singh, 1994) subscales showed significant between-group differences [*Irritability*, *F*_(4,205)_ = 3.14, *p* = 0.028; *Lethargy/Social Withdrawal*, *F*_(4,205)_ = 3.52, *p* = 0.017; and *Hyperactivity*, *F*_(4,205)_ = 3.99, *p* = 0.009]. *Post hoc* analysis showed significant *Irritability* scores decrease post-18 TMS vs. baseline (-6.01 ± 2.02, from 12.39 ± 9.63 to 6.38 ± 4.59, *p* = 0.029, **Figure 7B** left), as well as *Lethargy/Social Withdrawal* scores decrease (-5.08 ± 1.78, from 11.50 ± 8.09 to 6.42 ± 5.91, *p* = 0.040) along with decreased *Hyperactivity* scores (-7.34 ± 2.81, from 18.09 ± 12.74 to 10.75 ± 9.22, *p* = 0.049, **Figure 7B** right).

## Discussion

Results of our exploratory clinical research study showed significant changes from baseline in functional measures of performance and behavioral symptoms rating during an oddball task in children with autism following 18 sessions of rTMS treatment. Several functional measures showed a difference from baseline and waitlist in RT and ERP variables after 12 sessions of rTMS, but only a few of them reached statistical significance post-6 session rTMS course. It should be noted, as a limitation of this study, that in this particular group rTMS was administered only over the left DLPFC and this could have biased the outcome. For the purposes of our study, demonstration of preference and advantages of 18 sessions of rTMS applied bilaterally over DLPFC was the most striking finding.

Among the most notable ERP changes in our study are the attenuated amplitude and longer latency of posterior P100 and anterior N100 ERPs to all types of stimuli, with post-TMS changes being more pronounced in response to non-target Kanizsa stimuli. Parietal P3b in response to targets was found to be prolonged without any amplitude change, while latency of P3b to non-targets was shorter in all TMS groups, more so in the 18 TMS group. At the frontal topography, latency of N100 was prolonged to targets and shortened to non-target items in the TMS groups, while remaining unchanged to all stimuli in the waitlist group. Frontal P3a was characterized by a shorter latency to non-target Kanizsa distracters and standards after TMS treatment but not after wait period.

Several behavioral symptoms of autism showed improvement post-TMS according to caregivers’ reports (RBS-R, Bodfish et al., 1999; ABC, Aman and Singh, 1994). Most notable were a decrease of *T*-score of the RBS-R after 18 sessions of rTMS, along with decreased irritability, lethargy/social withdrawal and hyperactivity rating scores of the ABC questionnaire. Changes in the aforementioned measures of aberrant behavior and repetitive and stereotyped behaviors were comparable to those found in our prior studies where similar parameters and length of TMS intervention were used in children and adolescents with autism (Sokhadze et al., 2014a,b).

In order to better understand the effects of rTMS-based neuromodulation, it is important to briefly review differences in RT, ERN/Pe and stimulus-locked ERPs between children with ASD and neurotypical controls. In a series of studies (Sokhadze et al., 2009b, 2010b, 2013) using different variations of three-stimulus oddball tasks we reported delayed latencies of early and late ERP components to non-target items without significant differences in amplitude characteristics in response to targets in children with autism as compared to neurotypical peers. We interpreted our finding as indicative of a deficient ability to recognize the distinction between task-relevant and task-irrelevant stimuli.

In other studies, using a similar oddball paradigm (Sokhadze et al., 2009b, 2010b; Baruth et al., 2010c), we found, in accordance with the majority of results of RT tasks in children with autism, that differences in motor responses during oddball tests were observed in accuracy measures and in post-error RT adjustments rather than in reaction time (Baruth et al., 2010c; Sokhadze et al., 2012b, 2017). We interpreted these deficits in post-error response speed correction, as well as impulsive perseverative fast post-error response, as a manifestation of an abnormality of error monitoring and correction function (Sokhadze et al., 2010a, 2012b, 2014a,b, 2016, 2017). Deficient magnitude of ERN was reported by other ERP studies (Henderson et al., 2006; Bogte et al., 2007; Thakkar et al., 2008; Vlamings et al., 2008) with an indication that individuals with autism tend to exhibit reduced performance monitoring ability and often fail to adjust behavior after a committed error. This performance monitoring and error correction deficit reflects a lower sensitivity of error detection and a reduced erratic response correction capacity in ASD individuals. Most studies on ERN/Pe components could not find differences in the Pe measure between children with autism and neurotypical children (reviewed in Sokhadze et al., 2010a, 2012b, 2017). In our studies using an oddball paradigm, children with autism did not improve accuracy after committed errors and did not show normative post-error RT slowing (Sokhadze et al., 2012a,b). As a rule, in typical controls the performance on trials immediately following commission errors tend to improve as a result of a change in the speed–accuracy strategy. Implementation of this strategy results in a normative post-error RT slowing and less errors committed; a process dependent on executive control functioning. Impairment in error detection and adjustment of post-error performance in children with autism points at an executive control deficiency that may have negative consequences since effective error detection and correction function is necessary for adequate behavior adjustment (Sokhadze et al., 2010a, 2012b).

Since we are not aware of other studies where ERPs were used as outcomes of rTMS in children with ASD, we can only compare the findings of this study with our own prior preliminary studies (Sokhadze et al., 2009a,b, 2012a, 2014a,b; Baruth et al., 2010a,c; Casanova et al., 2012, 2014b). Most notable, our studies found that neuromodulation based on rTMS facilitated target recognition in reaction time tests. Quite important in this regard was the positive trends for changes in both P3a and P3b potentials and in the enhanced reactivity of these cognitive ERPs to target stimuli vs. non-target stimuli. The latter findings reflect enhanced discrimination of features determining specifics of the target illusory stimuli (i.e., Kanizsa square vs. Kanizsa triangle). A reduction in amplitude and latency of cognitive ERPs (P3a and P3b) to frequent standard non-Kanizsa and rare non-target Kanizsa figures post-TMS in children with autism, especially in the 18 TMS group as compared to the baseline and also waitlist group outcomes, can be considered as a sign of improved target discrimination. Prolonged latency of the frontal P3a to infrequent distracters at baseline test can be considered a potential biomarker of impaired orientation to novelty. In our study, TMS improvement of this measure represents an enhancement of this important integrative function.

Over-activation in the parietal and parieto-occipital cortex at the initial stages of unattended stimuli, along with delayed and prolonged activation of the frontal cortex at the later stages of attended stimuli processing has been reported in children with ASD in a similar visual oddball test with novel distracters (Sokhadze et al., 2010a,b, 2017). We interpreted these results as a manifestation of low selectivity in perceptual processing that leads to an over-processing of unattended stimuli at the later stages. Treatment using low frequency rTMS exerted positive effects on both early and late stages of signal processing and improved ability of less effortful differentiation of targets from non-target items.

The results of the current study indicate that rTMS, the effect being better expressed after 18 rTMS treatment, may facilitate cognitive top-down control and enhance target discrimination specifically during the processing of infrequent task-relevant and task-irrelevant illusory Kanizsa figures. It is noteworthy that the latency of P3b post-TMS was longer to targets and at the same time shorter to both rare non-targets and frequent standards. The P3b is the best studied cognitive component (Polich, 2003, 2007) and has been considered as a marker of task-relevance evaluation, memory-updating and individual trial processing closure (Picton, 1992). Most of the ERP studies in autism focused on outcomes of the endogenous cognitive potentials such as P3b (Courchesne et al., 1989; Ciesielski et al., 1990; Kemner et al., 1994, 1999; Jeste and Nelson, 2009) and to a lesser extent P3a (Townsend et al., 2001). This frontal cognitive potential in our prior studies was delayed but not significantly reduced in children with ASD as compared to neurotypical children (Sokhadze et al., 2009a,b, 2010b, 2012b). In the current study, the P3a component was also found to be prolonged.

Atypicality of exogenous ERP components in individuals with autism has been the object of few studies. Some of these studies have reported abnormalities of sensory perceptual processes, low selectivity both in visual and auditory modalities of stimulation, and most importantly deficits of cognitive control which negatively affects information integration processes (Belmonte and Yurgelun-Todd, 2003). The outcomes of the current study emphasize facilitated target discrimination and enhanced habituation to non-target stimuli post-TMS treatment, more pronounced in the 18 TMS group. Behavioral responses changes (e.g., RT, accuracy) are considered as a very robust measure in oddball tasks, and hence it was very important to find significant improvements in the accuracy of performance on tasks, restoration of normative post-error RT slowing, along with slower RT during commission errors (markers of impulsivity) following 18 session-long rTMS course. Outcomes of 12 session-long rTMS course had similar trends though some of the above indices did not reach the level of statistical significance. In general, our current study indicates improvements in attention, executive functions (e.g., performance monitoring), and enhanced irrelevant response inhibition following rTMS-based intervention in children with ASD. An important addition to our previous studies was the ability to compare the dosage effects of rTMS over different number of sessions.

Executive deficits have always been of interest in autism research (Ozonoff et al., 1991). Abnormalities in error detection, performance monitoring and response conflict resolution help explain many clinically relevant behavioral symptoms present in autism. These executive functions can be readily measured using response-locked ERPs, specifically ERN and Pe (Gehring et al., 1993; Carter et al., 1998; Van Veen and Carter, 2002; Mars et al., 2005; Arbel an Donchin, 2009; Arbel and Donchin, 2011). The ERN is a well-studied ERP with ties to response error processing (Carter et al., 1998; Falkenstein et al., 2000; Gehring and Knight, 2000; Van Veen and Carter, 2002; Davies et al., 2004; Huizinga et al., 2006). The ERN abnormalities, and more specifically attenuated amplitude or delayed latency of this measure, are interpreted as indices of error processing impairments (Ridderinkhof et al., 2004). In this context, it should be outlined that among the most important findings of the current study was a replication of the increase in ERN magnitude following TMS-based neuromodulation that was reported in our prior studies (Sokhadze et al., 2012b, 2014a,b; Casanova et al., 2014b). In these studies, we found enhanced ERN amplitude post-TMS without any Pe changes. This component is thought to reflect conscious assessment that an error was committed (Falkenstein et al., 2000; Nieuwenhuis et al., 2001; Overbeek et al., 2005).

In our preliminary trials using rTMS intervention in children with autism (Sokhadze et al., 2009a,b, 2010b; Baruth et al., 2010a,c) we found that most ERP changes were observed at the early phases of visual signal processing resulting in easier discrimination of targets in oddball task. Enhanced target recognition post-TMS intervention and facilitated pre-attentive inhibition of task-irrelevant stimuli resulted in a lesser load during processing of non-target during later stages. The present study reproduces the positive effects of rTMS on the early ERPs probably due to enhanced suppression of distracters leading to less effortful discrimination of targets from rare non-targets and frequent standards during performance in oddball task (Casanova et al., 2014b; Sokhadze et al., 2017).

Several limitations to the study should be mentioned. One of them is related to the frequency of rTMS sessions, as some of rTMS-based intervention in various psychiatric treatment studies used a more intensive schedule of stimulation (e.g., daily, twice or thrice per week). We selected a weekly regimen because it was better accepted by the families of our study participants. We believe that the effects of rTMS do not wash out in a week’s period, and based on our empirical observations led to good retention, while providing improvements in behavioral symptoms as well as functional outcomes. It is possible to suggest that the length of treatment course (e.g., 12 or 18 weeks) rather than the treatment frequency is one of the main factors of observed behavioral and ERP improvements in our trials in children with autism (Baruth et al., 2010c; Sokhadze et al., 2010b, 2012a, 2017; Casanova et al., 2012). It must be noted that the selected power of rTMS pulses was lower than motor threshold and total number of administered TMS pulses was relatively lower as compared to other established rTMS treatment protocols (e.g., rTMS for major depression). One of the safety related reasons of our intensity preference was the fact that we were among the first groups that started using rTMS in this particular population of children known to be predisposed to seizures.

An additional limitation of the study was the selection of a waiting list group as a form of a control condition instead of using a randomized clinical trial (RCT) design with participants being randomly assigned to either active or sham rTMS group. Custom-made sham coil and interface for double-blinding of TMS delivery are available in our lab, however, they do not mask the muscle contractions of the scalp. There are still several factors that can be investigated using waitlist group design rather than immediately progressing to a double-blind RCT with a sham TMS arm. One of the very important feasibility issues is the number of rTMS sessions and this particular question was addressed in our current study.

Among the limitations that should be considered is the fact that we only stimulated the left hemisphere in the 6 TMS group. This could serve as a confound when making between group comparisons, though all three groups had at least six rTMS treatments over the left DLPFC. Nevertheless, positive changes from the baseline in the 18 TMS group as compared to waitlist group serves to confirm the advantage of this particular dosage of low frequency rTMS over DLPFC for inducing improvements in functional measures of performance on cognitive task and in some of behavioral symptoms of the autism spectrum.

## Conclusion

Our comparative effectiveness study confirmed that intervention using low frequency rTMS over DLPFC in children with ASD has positive effects on performance in a visual oddball test and improves parental behavioral questionnaires scores reports. Among the most notable findings were improvements in ERP correlates of effective target discrimination, along with reduced excessive responsiveness to non-target task-irrelevant stimuli, accuracy of behavioral responses, and enhanced behavioral and ERN correlates of effective error detection, monitoring and correction function. Neuromodulation using rTMS significantly reduced rating scores of repetitive and stereotypic behaviors, as well as hyperactivity, social withdrawal and irritability scores according to social and behavioral evaluations. Based on observed changes of magnitude of dependent variables used in our comparative effectiveness study it is possible to conclude that longer courses of neuromodulation using low frequency rTMS offer significant improvements in measures of executive functions and behavior in children with ASD. The study also provides additional support to the statement that low frequency rTMS administered weekly over the DLPFC, with sufficient number of stimulation sessions, is a potentially effective treatment option targeting autistic symptoms such as executive function deficits, and aberrant/repetitive behaviors.

## Author Contributions

ES designed ERP test, analyzed ERP data, conducted rTMS stimulation, and participated in interpretation of data and manuscript preparation. EL and EC coordinated study, subjects recruitment, conducted part of statistical analysis, illustrations, and helped in manuscript preparation. DK consulted on all clinical questions related to autism spectrum disorder and participated in manuscript preparation. IO developed method for reaction time and other behavioral measures analysis and interpretation. AT consulted on clinical issues related to autism and other aspects of TMS application in psychiatry, participated in review of manuscript. MC designed approach for TMS application in autism treatment and research, developed theoretical framework of the study, actively participated in interpretation of data, and manuscript preparation for publication.

## Conflict of Interest Statement

The authors declare that the research was conducted in the absence of any commercial or financial relationships that could be construed as a potential conflict of interest.
